# Expression of MLL-AF4 or AF4-MLL fusions does not impact the efficiency of DNA damage repair

**DOI:** 10.18632/oncotarget.8938

**Published:** 2016-04-22

**Authors:** Julio Castaño, Ana B. Herrero, Aldeheid Bursen, Federico González, Rolf Marschalek, Norma C. Gutiérrez, Pablo Menendez

**Affiliations:** ^1^ Josep Carreras Leukemia Research Institute, Department of Biomedicine, School of Medicine, University of Barcelona, Barcelona, Spain; ^2^ Hematology Department, University Hospital of Salamanca, IBSAL, IBMCC (USAL-CSIC), Salamanca, Spain; ^3^ Institute Pharmaceutical Biology, Goethe-University, Frankfurt/Main, Germany; ^4^ IBEC - Institute for Bioengineering of Catalonia, Barcelona, Spain; ^5^ Institució Catalana de Recerca i Estudis Avançats (ICREA), Barcelona, Spain

**Keywords:** MLL.AF4, AF4.MLL, t(4;11), DSB, infant leukemia

## Abstract

The most frequent rearrangement of the human MLL gene fuses MLL to AF4 resulting in high-risk infant B-cell acute lymphoblastic leukemia (B-ALL). MLL fusions are also hallmark oncogenic events in secondary acute myeloid leukemia. They are a direct consequence of mis-repaired DNA double strand breaks (DNA-DSBs) due to defects in the DNA damage response associated with exposure to topoisomerase-II poisons such as etoposide. It has been suggested that MLL fusions render cells susceptible to additional chromosomal damage upon exposure to etoposide. Conversely, the genome-wide mutational landscape in *MLL*-rearranged infant B-ALL has been reported silent. Thus, whether MLL fusions compromise the recognition and/or repair of DNA damage remains unanswered. Here, the fusion proteins MLL-AF4 (MA4) and AF4-MLL (A4M) were CRISPR/Cas9-genome edited in the AAVS1 locus of HEK293 cells as a model to study MLL fusion-mediated DNA-DSB formation/repair. Repair kinetics of etoposide- and ionizing radiation-induced DSBs was identical in *WT*, MA4- and A4M-expressing cells, as revealed by flow cytometry, by immunoblot for γH2AX and by comet assay. Accordingly, no differences were observed between *WT*, MA4- and A4M-expressing cells in the presence of master proteins involved in non-homologous end-joining (NHEJ; i.e.KU86, KU70), alternative-NHEJ (Alt-NHEJ; i.e.LigIIIa, WRN and PARP1), and homologous recombination (HR, i.e.RAD51). Moreover, functional assays revealed identical NHEJ and HR efficiency irrespective of the genotype. Treatment with etoposide consistently induced cell cycle arrest in S/G2/M independent of MA4/A4M expression, revealing a proper activation of the DNA damage checkpoints. Collectively, expression of MA4 or A4M does neither influence DNA signaling nor DNA-DSB repair.

## INTRODUCTION

The *mixed-lineage leukemia* (*MLL*) gene fuses to generate chimeric genes with 80 partners in human leukemia [1]. Infant pro-B acute lymphoblastic leukemia (B-ALL) harboring the fusion MLL-AF4 (MA4) represents a rare leukemia, associated with very brief latency and dismal prognosis, raising the question of how this disease evolves so quickly [2]. Epidemiological and genetic studies support the contention that the *in utero* origin of MA4 in infant B-ALL may be the result of transplacental exposures during pregnancy to quinone-based chemicals or dietary flavonoids [3–5]. This parallels the origin of therapy-related “secondary” leukemias harboring *MLL*- rearrangements [4, 6, 7] and is supported by the finding that *MLL* gene rearrangements can be induced *in vitro* and *in vivo* in hematopoietic stem/progenitor cells (HSPCs) at different ontogeny stages by etoposide, a topoisomerase-II inhibitor commonly used in chemotherapy regimens [8–11].

Exposure to environmental agents may represent a potential etiological driver in *MLL*-rearranged leukemia. *MLL-* rearrangements are the consequence of mis-repaired DNA double strand breaks (DNA-DSBs) [12, 13]. Alternatively, they might be due to defects in the DNA damage response (DDR) after chronic exposure to topoisomerase-II poisons (etoposide, bioflavonoids, and pesticides) or even irradiation in early HSPCs [3–5, 7, 14]. Additionally, overexpressed MLL fusions have been shown to render cells more susceptible to additional chromosomal damage upon exposure to etoposide, suggesting that expression of MLL fusion proteins not only transform cells but also compromise the recognition and/or repair of DNA damage [15]. Contrasting this view are the recent findings deciphering a silent mutational genome-wide landscape in *MLL*-rearranged infant B-ALL [16–19]. Therefore, the genomic stability observed in *MLL*-r infant leukemia poses the question of whether or not the recognition and repair of DNA damage is influenced by MLL fusions. Here, we have attempted to address this question by exploiting a syngeneic human cell system in which a single copy of the fusion protein MA4 or A4M was CRISPR/Cas9-genome edited in the AAVS1 locus of HEK293 cells as a model to study MLL fusion-mediated DNA-DSB formation and repair.

## RESULTS

### CRISPR/Cas9-mediated insertion of MA4 and A4M into AAVS1 safe harbor

**Figure 1:**
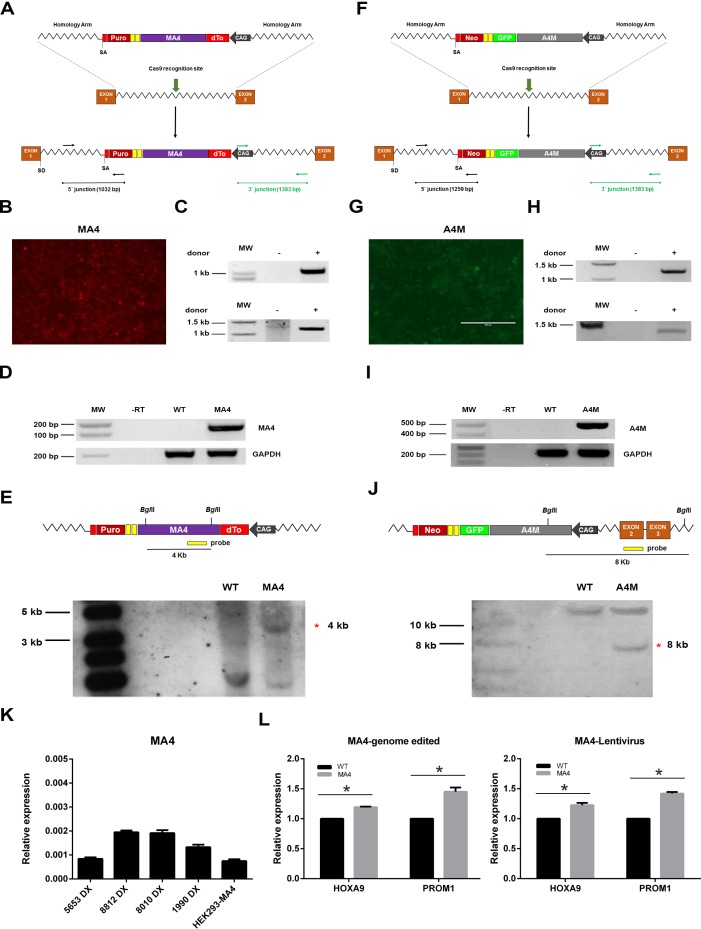
CRISPR/Cas9-mediated generation of syngeneic HEK293 cells expressing a unique copy of MA4 or A4M in the AAVS1 safe harbor **A.**, **F.** Schematic representation of the donor vector used for insertion of the dTo-MA4 **A.** or A4M-GFP **F.** cassette into the AAVS1 locus. dTo, dTomato fluorescent protein; SD, splice donor; SA, splice acceptor; CAG, CMV early enhancer/chicken β actin promoter. Black (5′; junction) and green (3′; junction) arrows depict genomic location of primers used to confirm targeted integration. **B.**,**G.** Representative images of dTo-MA4- and A4M-GFP-expressing HEK293 cells after puromycin or G418 selection, respectively. **C.**,**H.** Targeted integration analysis of MA4 and A4M into the AAVS1 locus by PCR using primers specific for the 5ʹ (top panels) and 3ʹ (bottom panels) integration junctions. **D.**,**I.** RNA expression of MA4 **D.** and A4M **I.** in antibiotic-selected cells. **E.**, **J.** Homologous recombination confirmed by southern blot analysis after BglII digestion of genomic DNA from puromycin/G418-resistant clones using a MA4 probe **E.** or an AAVS1 exon2 probe outside the targeting construct **J.**. A 4Kb band represents a targeted integration of MA4 in PPP1R12C. The 8kb band corresponds to the targeted integration of A4M in PPP1R12C. Untargeted allele gives a 12Kb band **J.**. **L.** qPCR of the MA4 targets HOXA9 and PROM1 is comparable between HEK293 cells ectopically expressing MA4 upon CRISPR/Cas9-mediated genome edition (left panel) or lentiviral transduction (right panel) *p < 0.05, compared to *WT*. **K.** MA4 qPCR comparing B-ALL patients and HEK293-MA4.

To address whether the recognition and repair of DNA damage is regulated by MA4 and A4M expression, a single copy of each fusion was CRISPR/Cas9-genome edited in the AAVS1 locus of HEK293 human cells. Two donor vectors harboring dTo-MA4 or A4M-GFP cassettes under the transcriptional control of CAG promoter, and flanked by AAVS1 homology arms were generated (Figure 1A, 1F). These vectors contain a promoterless puromycin (Figure 1A) or neomycin (Figure 1F) cDNA preceded by a splice acceptor (SA) site and a translational self-cleaving 2A sequence. Successful Cas9-targeted insertion into AAVS1 locus confers transcription of both puromycin and neomycin cassettes under the control of the ubiquitously expressed *PPP1R12C* promoter, thus allowing the antibiotic selection of the targeted cells. A guide-RNA directed to AAVS1 locus was used to enhance the gene targeting efficiency. After antibiotic selection, many antibiotic-resistant clones were isolated and MA4- and A4M-targeted clones were dTo+ and GFP+, respectively (Figure 1B, 1G). PCR analysis using primers specific for 5ʹ and 3ʹ integration junctions of the AAVS1 locus showed proper genomic integration for MA4 and A4M, respectively (Figure 1C, 1H). RT-PCR demonstrated specific RNA expression of MA4 and A4M transcripts in the targeted cells (Figure 1D, 1I). Importantly, southern blot analysis further confirmed a single copy correct integration of the donor vectors (MA4 and A4M cassettes, Figure 1E, 1J). Importantly, the expression level of MA4 in MA4-edited HEK293 cells was comparable with that one of four independent primary t(4;11)+ pediatric B-ALL (Figure 1K). Furthermore, the MA4 target genes *HOXA9* and *PROM1* were similarly upregulated upon either CRISPR/Cas9- or lentiviral-mediated integration/expression (Figure 1K). Together, CRISPR/Cas9-genome edited isogenic cells were developed allowing us to address whether MA4 and A4M facilitate/impair DNA-DSB formation/repair.

### Expression of either MA4 or A4M does not affect DNA-DSB formation nor repair

**Figure 2:**
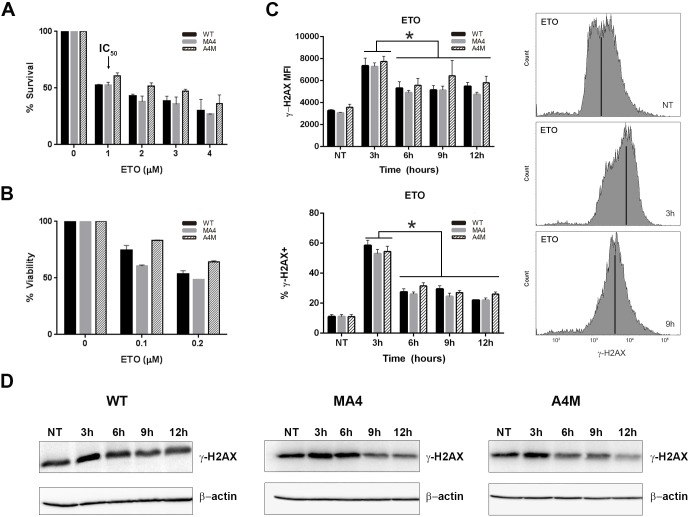
Kinetics of γH2AX loss after etoposide treatment **A.** Cell viability measured by MTT assay for increasing concentrations of etoposide (*n* = 3). The IC_50_ is 1 μM irrespective of the genotype. **B.** Clonogenic survival measured 12-days after etoposide treatment revealing no differences between genotypes (*n* = 3). In A and B data is expressed as percentage of cell death relative to untreated control. **C.** Time course of γH2AX MFI (top left) and % of γH2AX+ cells (bottom left) at the indicated time points after 1μM etoposide pulse (*n* = 3). Representative flow cytometry histograms of γH2AX staining in non-treated cells (NT) or cells 3h and 9h after 1μM etoposide pulse (right panel). **D.** Phospho-γH2AX western blot at different time points after etoposide treatment. β-actin was used as loading control. *p < 0.05, compared to NT.

The most deleterious form of DNA lesions are DSBs. Mis-repair of DSBs leads to either cell death or genome rearrangements [20]. To investigate whether MA4 or A4M impact DNA-DSB formation and/or repair, we first analyzed the effect of etoposide, a *bona fide* topoisomerase-II poison, on cell proliferation and clonogenic survival. MTT proliferation assays revealed identical IC_50_ (~1μM) for etoposide, irrespective of the investigated genotype (Figure 2A). Similarly, clonogenic survival measured 12-days after etoposide treatment revealed no differences among genotypes (Figure 2B). To analyze the kinetics of DSB formation and DNA repair, we monitored the phosphorylation of Ser139-H2AX (γH2AX) [21] every 3h after treatment with 1μM etoposide. γH2AX signal was quantified by flow cytometry in *WT*, MA4- and A4M-expressing cells. γH2AX intensity (MFI) and the proportion of γH2AX+ cells reached their maximum 3h after etoposide treatment and started to fall over the next 12h, again with identical kinetics irrespective of the investigated genotype (Figure 2C). To confirm the flow cytometry data, γH2AX expression was analyzed by Western Blot. γH2AX peaked 3h after etoposide treatment and then fall over the next 12h, with similar kinetics for *WT*, MA4- and A4M-expressing cells (Figure 2D).

**Figure 3:**
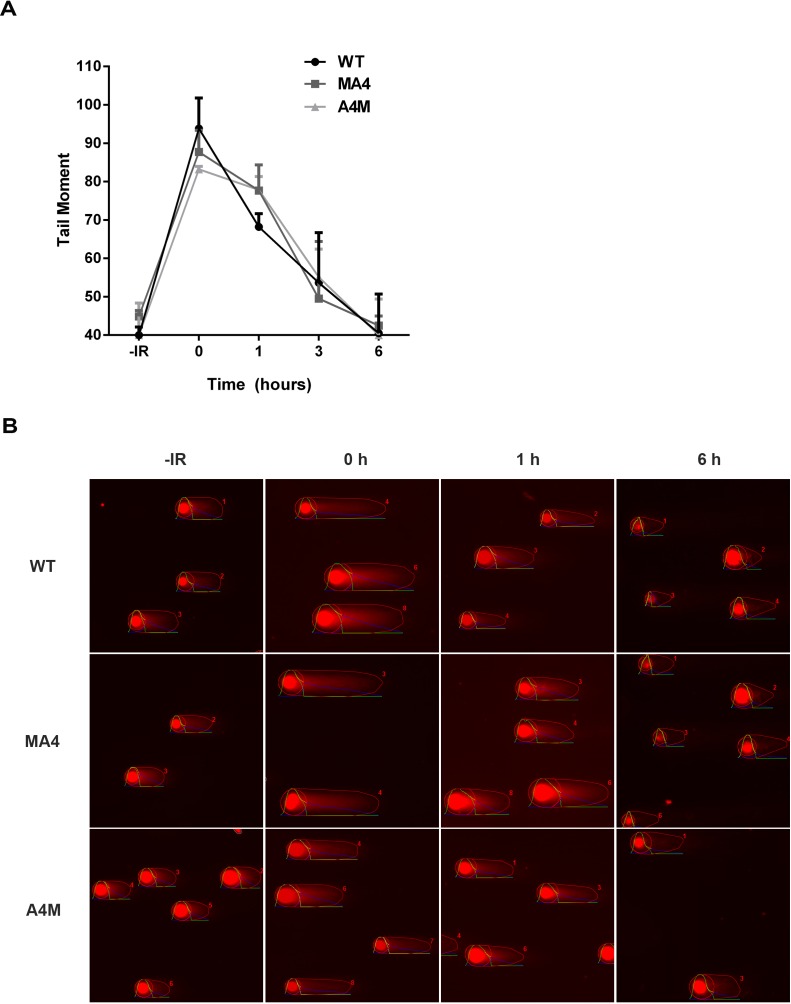
Analysis of DSB repair by neutral comet assay **A.** *WT*, MA4 and A4M cells were irradiated with 40Gy and the mean tail moment calculated using the OpenComet software. Data represent values of at least 75 comets in each of two independent experiments. **B.** Representative images of the comet assay.

Next, we analyzed the repair kinetics of IR-induced DSBs using the neutral comet assay. Similar to the kinetics of γH2AX loss, no differences were found in the kinetics of DSBs repair between MA4, A4M and *WT* cell lines (Figure 3A, 3B). For all the genotypes most of the DNA damage was repaired 6h after IR, despite the high irradiation dose given (40 Gy) (Figure 3A, 3B). These data indicate that MA4- and A4M-expressing cells are capable of repairing most of both etoposide- and IR-induced DSBs similar to *WT* syngeneic cells.

**Figure 4:**
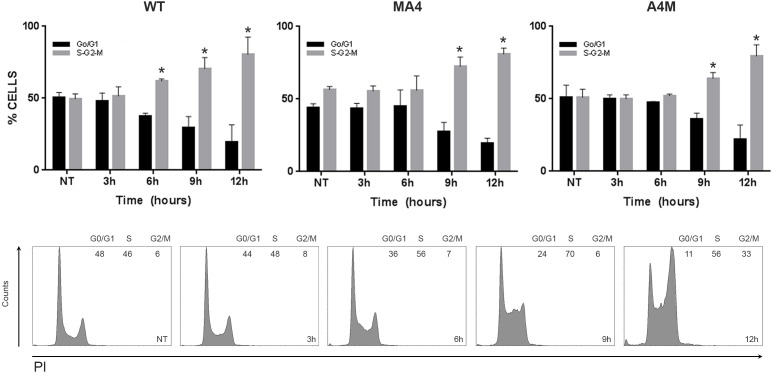
Cell cycle distribution assessed at the indicated time points after etoposide pulse Top panels represent the % of cells in G0/G1 *vs* S/G2/M cell cycle phases analyzed by FACS for the indicated genotypes (*n* = 3). Bottom panels are the corresponding flow cytometry histograms. Data represents mean±SD of three independent experiments *p < 0.05.

Cell cycle checkpoints are activated following induction of DSBs, providing time for the removal of the DNA damage [20]. In fact, etoposide induces G2/M checkpoints [22] which efficiently retain cells in G2 until they partially repair DSBs. Cell lines consistently showed a G2/M arrest 9h after etoposide treatment which was even more pronounced after 12h (Figure 4). This G2/M arrest was independent of MA4 and A4M expression, revealing a proper activation of the DNA damage checkpoints.

### MA4 and A4M expression does not regulate either NHEJ or HR repair pathways

**Figure 5:**
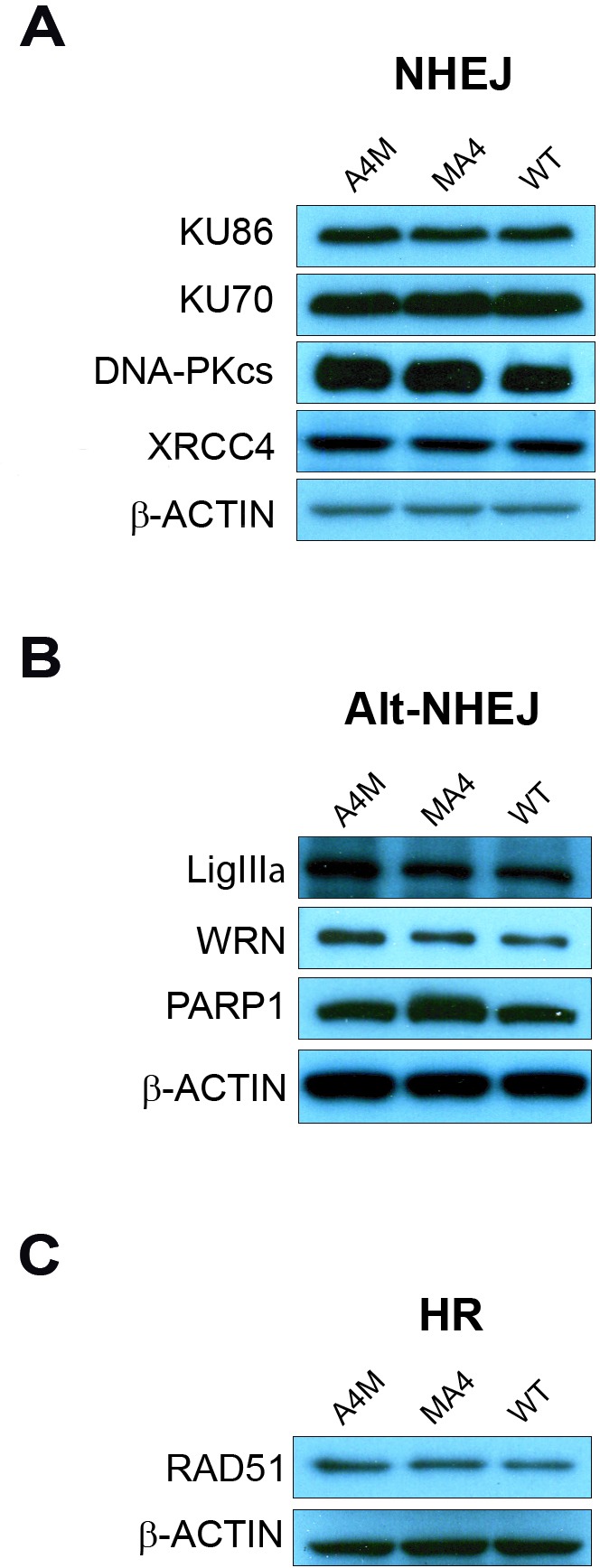
Western blot analysis of proteins involved in DSB repair Proteins involved in the classical NHEJ pathway **A.**, Alt-NHEJ proteins **B.** and the HR protein Rad51 **C.** are shown.

To further confirm that MA4 and A4M do not affect DSB repair, we analyzed the steady-state levels of master proteins involved in the main pathways of DSB repair: NHEJ, HR and alternative NHEJ (Alt-NHEJ) [23]. Western blot analysis was performed in *WT*, MA4 and A4M cell lines (Figure 5). No differences were observed between *WT*, MA4- and A4M-expressing cells for the expression of proteins involved in NHEJ (KU86, KU70, DNA-PK_cs_ and XRCC4, Figure 5A), Alt-NHEJ (LigIIIa, WRN and PARP1, Figure 5B), and HR (RAD51, Figure 5C).

**Figure 6:**
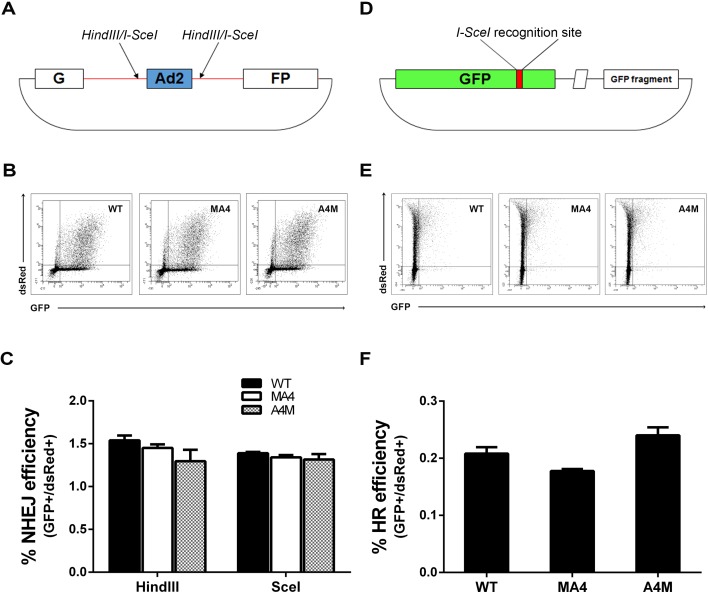
Analysis of NHEJ and HR in WT, MA4 and A4M-expressing cells **A.** Schematic map of pEGFP-Pem1-Ad2 and pHR plasmids [38]. **B.** *WT*, MA4 and A4M cells were transfected with *Hind*III- or *Sce*I-digested plasmid together with pDSRed2-N1 to normalize transfection efficiencies. GFP *vs* DsRed flow cytometry data is shown 24 h after transfection. **C.** Percentage of NHEJ of *Hind*III- or *Sce*I-digested plasmid for the three genotypes (*n* = 3). NHEJ efficiency was calculated as the ratio of GFP+/DsRed+ cells. **D.** Reporter plasmid used for detection of HR. **E.** *WT*, MA4 and A4M cells were transfected with *Sce*I-digested HR plasmid together with pDSRed2-N1. GFP *vs* DsRed flow cytometry data is shown 48 h after transfection. **F.** The ratio of GFP+/DsRed+ was used as a measure for HR repair efficiency. Data represents mean±SD of three independent experiments.

To further investigate the efficiency of NHEJ and HR, functional assays were conducted by measuring the ability of the target cells to re-circularize *Hind*III*- or Sce*I-digested pEGFP-Pem1-Ad2 NHEJ plasmid (Figure 6A) and *Sce*I-digested HR reporter plasmid (Figure 6D). Successful re-ligation by the cell of enzyme-digested NHEJ or HR plasmids restores the expression of GFP. Thus, the percentage of GFP+ cells is a *bona fide* indicator of successful repair of DSBs by either NHEJ or HR. pDSRed2-N1 circular plasmid was always used to correct the transfection efficiency (Figure 6B, 6E). The proportion of GFP+ cells was almost identical in *WT*, MA4 and A4M cell lines, indicating identical NHEJ and HR efficiency irrespective of the investigated genotype (Figure 6C, 6F). Collectively, expression of either MA4 or A4M does not seem to influence either DNA signaling or DNA damage repair.

## DISCUSSION

*MLL*-rearrangements are common in *de novo* infant acute leukemia and in therapy-related AML secondary to treatment with topoisomerase-II inhibitors (i.e. etoposide) [6]. Similar to IR-driven damage, topoisomerase-II poisons induce DSBs which are the most deleterious form of DNA lesions in living organisms. Mainly cells use the NHEJ-mediated DNA repair mechanism in G0 and G1 [24], while using HR in replicating cells or G2 where a proper template becomes available. Depending on the amount of DSBs in a given cell, DSBs may compromise cell viability and will lead to cell death [20]. Assuming the cells remain usually in G0/G1, NHEJ-mediated DNA repair processes are more important. However, this type of repair frequently end in small deletion, duplications or inversions [12, 13]. Therefore, *MLL*-rearrangements can still be assumed as a consequence of mis-guided DNA repair [25]. In addition, exposure to topoisomerase-II poisons such as etoposide and bioflavonoids will increase the rate of DSBs, and thus, increase the probability of misguided DNA repair, resulting in chromosomal translocations [3–5, 7, 14]. Noteworthy, MLL-ENL fusions have been proposed to confer further susceptibility to chromosomal damage upon exposure to etoposide, suggesting that expression of MLL fusions not only transform cells but might also compromise the recognition and/or repair of DNA damage [15]. Similarly, MLL fusions have been found to alter cell cycle dynamics [26, 27] and to suppress DNA damage induced apoptosis mediated by TP53 [28]. Furthermore, specific transcriptional programs, including those involved in DDR, are frequently deregu­lated by various oncogenic transcription factors and chimeric genes. In contrast, recent findings revealed a silent mutational genome-wide landscape in *MLL*-rearranged infant B-ALL [16–19]. The genomic stability observed in *MLL*-r infant leukemia poses then the question of whether or not MLL fusions render cells more vulnerable to further DNA damage and mutations through deregulation of DDR signaling. Here, we harnessed CRISPR/Cas9-based genome editing to develop a system harboring either MA4 or A4M in the AAVS1 safe harbor of HEK293 cells. This model was useful to explore the influence of MLL fusion proteins on the amount of inducible DNA DSBs and the fidelity of the subsequent DNA repair process. The established cell culture model is unique because of the following: i) it is a syngeneic cellular model, ii) cells carry a single copy of each MLL fusion, and iii) the integration in a safe harbor ensures stable expression of both MA4 and A4M.

Repair kinetics of etoposide- and IR-induced DSBs was identical in *WT*, MA4- and A4M-expressing cells, as revealed by flow cytometry, by immunoblot for γH2AX and by the comet assay. In addition, functional assays revealed identical NHEJ and HR efficiency irrespective of the investigated genotype. These results indicate that although topoisomerase-II poisons may induce *MLL* -rearrangements, these MLL chimeric proteins do not subsequently render cells more vulnerable to further DNA damage. In other words, MA4- or A4M-expressing cells that have survived exposure to genotoxic etoposide are equally likely to carry further DSBs than *WT* cells. Molecular cytogenetic techniques were not used to analyze metaphases/interphases of cells exposed to etoposide. However, the absence of persistent DSBs and the similar NHEJ and HR repair efficiencies make specific chromosomal changes (structural and numerical alterations) or double minutes unlikely to occur since persistent DSBs must precede further DNA damage. One limitation of our study is the use of the HEK293 instead of hematopoietic cells. Of note, however, any hematopoietic immortalized cell line would carry important basal genomic instability similar to human kidney-derived 293 cells. The ideal scenario to address this question would rely on the generation of CRISPR/Cas9-genome edited primary CD34+ progenitors. However, reporter/antibiotic selection of primary CD34+ progenitors carrying the functional t(4;11) is challenging [29–31], and using syngeneic cells is an asset to reduce (epi)-genetic variability among target cells.

Cell cycle checkpoints are activated following induction of DSBs, providing time for the removal of the DNA damage [20]. Etoposide induces G2/M checkpoints which efficiently retain cells in G2 until they partially repair DSBs [22], and early G2/M checkpoint failure has been proposed as a molecular mechanism underlying etoposide-induced chromosomal aberrations [32]. A cell cycle arrest in G2/M was achieved after etoposide treatment irrespective of MA4 and A4M expression, revealing a proper activation of the DNA damage checkpoints. A recent *in vivo* study has reported that rearrangements of the *MLL* gene can only occur when cooperating defects in the DDR are in place. For instance, defective ATM, CHK2 and p53 signaling bypasses arrest of cells in G2/M phase, thus limiting the time for the cells to repair the damage before continuing to divide [11, 32]. Because syngeneic cell lines were used it cannot be rule out that parallel or downstream insults cooperating with MLL fusions are also required to render further DNA damage vulnerability.

It has been very recently demonstrated that AML driven by repressive transcription factors, including AML1-ETO and PML-RARα are sensitive to poly (ADP-ribose) polymerase (PARP) inhibition, due to suppressed expression of HR-associated genes and impaired DDR associated to prevention of binding of KU proteins to DNA ends in NHEJ [33, 34]. In contrast, *MLL*-driven leukemias are proficient in DDR and insensitive to PARP inhibition. We found that MA4 and A4M do not regulate the expression of master proteins involved in the DNA repair pathways NHEJ, Alt-NHEJ and HR. Together, exposure to etoposide induces MLL fusions and preleukemic clone emergence but does not seem to facilitate the rapid acquisition of further mutations/DNA damage accelerating the clonal evolution to frank malignancy.

## MATERIALS AND METHODS

### Plasmids construction

pSpCas9(BB)-2A-GFP (PX458) plasmid was a gift from Prof. Feng Zhang (Addgene plasmid #48138). Guide RNA against AAVS1 intron 1 was constructed using the primers AAVS1 Fw 5ʹ-CACCGGGGCCACTAGGGACAGGAT-3ʹ and AAVS1 Rv 5ʹ-AAACATCCTGTCCCTAGTGGCCCC-3ʹ [35]. pAAVS1.SA-2A-Puro-pA donor vector was obtained from Addgene (plasmid #22075). pAAVS1.SA-2A-Neo-pA was made by replacing puro gene from pAAVS1.SA-2A-Puro-pA using *Xho*I and *Mfe*I restriction sites. CAG promoter was PCR-amplified and cloned into both donor vectors using *Not*I/*Pac*I restriction sites. A4M-GFP-*Sfi*I and dTo-MA4-*Sfi*I cassettes were subcloned in the Neo and Puro donor vector, respectively, by introducing in the donor vector a linker with two *Sfi*I restriction sites (Figure 1A, 1F).

### Cell culture, transfection and antibiotic selection

Human embryonic kidney 293 cells were maintained in Dulbecco's modified medium (DMEM; Gibco) supplemented with 10% fetal bovine serum (FBS, Sigma), 1X GlutaMAX and 1% penicillin/streptomycin solution (Gibco), at 37°C and 5% of CO_2_. For gene editing experiments, 90% confluent 293 cells were transfected in 6-well dishes with 1 μg pSpCas9 and 1 μg donor vector using Fugene HD transfection reagent (Roche) following manufacturer's instructions. Two days after transfection, cells were selected with 1 μg/ml puromycin or 1 mg/ml G418 (Sigma).

### PCR detection of targeted homologous recombination

In order to verify the CRISPR/Cas9-mediated integration in the AAVS locus, genomic PCR was performed using Taq DNA polymerase (Invitrogen) according to the manufacturer's instructions. To amplify the 5′; junction, two pairs of primers were designed inside the donor cassette (in the puro or neo gene) and outside the 5′; homologous recombination (HR) region. Primers in the CAG promoter and outside the 3′; HR region were used to amplify the 3′; junction. Primers used are detailed in Table 1S.

### RNA extraction, cDNA synthesis, PCR and qPCR

Total RNA was extracted using the RNAqueous kit (Ambion). Complementary DNA was synthesized using SuperScript III reverse transcriptase (Invitrogen). cDNA was used for conventional (MA4, A4M, GAPDH) and quantitative (MA4, HOXA9, PROM1) PCR. Conventional PCR was performed using Taq polymerase (Invitrogen) and quantitative PCR using Power SYBR^®^ Green PCR (Life Technologies) and a CFX384 Touch™ Real-Time PCR Detection System (BioRad). 2(−ΔCT) method was used to calculate the expression of the target mRNAs. Primers used are detailed in Table 1S.

### Southern blot

Ten μg of genomic DNA extracted from HEK293 cells was digested with *Bgl*II and separated on a 0.8% agarose gel by electrophoresis. The DNA was transferred onto a PVDF membrane and hybridized at 65°C overnight with a digoxigenin-dUTP-labeled probe. Primers used for probes are detailed in Table 1S.

### Immunoblot

10^6^ cells were washed with PBS and resuspended in RIPA lysis buffer (Santa Cruz Biotechnology (SCBT)) containing protease inhibitors (Roche) or 2X Laemmli Sample buffer. Protein concentration was measured using the Bradford assay (BioRad) and samples were heated to 100ºC for 5 min. Protein samples (20 μg/lane) were subjected to SDS-PAGE and transferred to PVDF membrane (BioRad). After blocking in T-TBS buffer plus 5% bovine serum albumin, membranes were incubated with anti-human antibodies. The following primary antibodies were used: anti-phospho-Histone H2AX (1:1000) (Ser139 clone JBW301, Millipore), anti-KU70 (1:12000) (A9, mouse, Santa Cruz Biotechnology), anti-KU86 (1:2000) (S10B1, mouse, Santa Cruz Biotechnology (SCBT)), anti DNA-PK_cs_ (1:1000) (rabbit, Abcam), anti-XRCC4 (1:1000) (A7, mouse, SCBT), anti-DNA ligase IIIα (1:1000) (1F1, mouse, Gene Tex), anti-WRN (1:1000) (H300, rabbit, SCBT), anti-PARP1 (1:1000) (rabbit, Calbiochem-Merck), anti-RAD51 (1:2000) (Rabbit, SCBT), and anti-β-Actin (1:100000) (mouse, SCBT). Horseradish peroxidase-linked donkey anti-rabbit or anti-mouse antibodies (SCBT) were used as secondary antibodies at 1:5000 dilution. Immunoblots were incubated for 1h at RT and developed using ECL detection reagents (Amersham, Piscataway, NJ).

### MTT assay

The effect of etoposide on cell proliferation was assessed by MTT assay (*n* = 3). Cells were seeded in triplicate in 96-well plates at 2×10^5^ cells/well. After 24 h, etoposide (or vehicle) was added at increasing concentrations (0-4 μM) for 3h. After a PBS wash, fresh medium was added and cells were incubated for 3 days. Then, 1 mg/ml MTT reagent (Sigma) was added and incubated for 3h. The absorbance was measured at 570 nm on an ELISA plate reader (Infinite 200, TECAN).

### Clonogenic survival assays

For clonogenic survival assays, duplicates of 2000 cells for each condition were seeded in 100 mm dishes. After 24 h, cells were treated with increasing concentrations of etoposide (or vehicle) for 3h. Cells were then PBS-washed, fed with fresh medium and maintained for 12 days. Cells were fixed and stained for CFU scoring with crystal violet solution (0.5% crystal violet in 20% ethanol).

### Flow cytometry analysis of γH2AX and cell cycle distribution

Half-million cells were seeded 24 h before treatment with 1μM etoposide (or vehicle) for 3h. Cells were then PBS-washed and harvested at the indicated time points following drug removal, fixed in cold 70% ethanol, and stored at −20ºC for 48 h. Cells were then stained with a mouse anti-human anti-phospho γH2AX antibody (Millipore) followed by an anti-mouse-AlexaFluor-647 secondary antibody (Cell Signaling). Subsequently, cells were suspended in propidium iodide (PI) buffer containing 5 μg PI and 100 μg/mL RNAase. Cell cycle distribution was analyzed on a FACSCanto-II cytometer using the FACSDiva software to discriminate among resting (G0/G1) and cycling cells (S-phase and G2/M) [36].

### Non-homologous end-joining (NHEJ) and homologous recombination (HR) assays

For these experiments HEK293T cells expressing either doxycycline-inducible MA4 or A4M were employed [37]. NHEJ and HR assays were performed as previously described [38]. Briefly, for NHEJ experiments 5×10^5^ cells were doxycycline-induced (1 μg/μl) for 24 h and then transfected using Fugene HD transfection reagent (Roche) with 0.2 μg of the normalizer plasmid pDSRed2-N1 and 0.5 μg of linearized pEGFP-Pem1-Ad2 [39]. GFP and DsRed fluorescence were measured 24 h later using a FACSAria cytometer. A total of 50000 cells were analyzed. For HR assays, 2 μg of the HR reporter construct were co-transfected together with 0.2 μg of pDsRed-N1 using Fugene. GFP+ and DsRed+ cells were quantified by flow cytometry 48 h after transfection. NHEJ and HR efficiency was calculated as the ratio of GFP+/DsRed+ cells [38].

### Comet assay

Cells were irradiated with 40 Gy of ionizing-radiation (IR) and were collected at different time points and processed for neutral comet assay as described previously [38]. Cell density was adjusted to 10^5^ cells/ml in ice-cold PBS and mixed with LMAgarose (Trevigen, Gaithersburg, MD) at 37°C at a ratio of 1:10 (v/v). Cell suspensions (25 μl) were immediately transferred onto CometSlide slides (20-well slides) and placed at 4°C for 10 min. Slides were submerged in N1 lysis solution [38] and incubated overnight at 37°C in the dark. After rinsing in N2 buffer, slides were subjected to electrophoresis in N2 solution for 30 min at 1V/cm. Cells were stained with ethidium bromide and analyzed with a fluorescence microscope (Zeiss Axioplan 2) equipped with a Hamamatsu Orca-EC camera. Images were obtained using Openlab software. At least 75 images per sample and experiment were analyzed. Tail moment was determined by the OpenComet software.

### Statistical analysis

Data are expressed as mean±sd of independent experiments. Statistical comparisons were performed using either paired or unpaired Student *t*-test (GraphPad Prism software), as corresponding. Statistical significance was defined as a *p*-value < 0.05.

## SUPPLEMENTARY MATERIAL TABLE


