# Supplementary material for: Expression of MLL-AF4 or AF4-MLL fusions does not impact the efficiency of DNA damage repair
Source: Oncotarget. 2016 Apr 22;7(21):30440–52. doi: 10.18632/oncotarget.8938 (PMC5058691; doi:10.18632/oncotarget.8938)
Supplement: Supplementary file 1 [file oncotarget-07-30440-s001.pdf]

Expression of MLL.AF4 or AF4.MLL fusions does not impact the efficiency of DNA damage repair

Supplementary Material

Table S1: Primers used in this study for PCR and Southern Blot.

MLL.AF4 Fw	5'-CCCAAAACCACTCCTAGTGAG-3'	qPCR
MLL.AF4 Rv	5'-ACTGTCACTGTCCTCACTGTCA-3'	
AF4.MLL Fw	5'-GTTGCAATGCAGCAGAAGCC-3'	
AF4.MLL Rv	5'-CAGGGTGATAGCTGTTTCGG-3'	
HOXA9 Fw	5'-AAGACCGAGCAAAAGACGAG-3'	
HOXA9 Rv	5'-GGGTGAGAGAAGGGAGAAGG-3'	
PROM1 Fw	5'-CTTTCTCCTGCCTCCCGC-3´	
PROM1 Rv	5'-TTTATGACCCGGCTTCTGGG-3'	
GAPDH Fw	5'-GCACCGTCAAGGCTGAGAAC-3'	
GAPDH Rv	5'-AGGGATCTCGCTCCTGGAA-3'	
Junction 5' Fw	5´-CTGCCGTCTCTCTCCTGAGT-3´	Homologous Recombination
Neo Rv	5´-CGTGGCCAGCCACGATAG-3´	
Puro Rv	5´-GTGGGCTTGTACTCGGTCAT-3´	
CAG Fw	GCTCACCTCGACCATGGTAAT-3´	
Junction 3' Rv	5´-GAATCCACCCAAAAGGCAGC-3´	
MA4 cDNA Fw	5'-CGCCTCAGCCACCTACTACAG-3'	Southern Blot
MA4 cDNA Rv	5'-GCGGCCATGAATGGGTC-3'	
AAVS exon 2 Fw	5'-ACAGGTACCATGTGGGGTTC-3'	
AAVS exon 2 Rv	5'-CTTGCCTCACCTGGCGATAT-3'	
